# Predictors of 72-h unscheduled return visits with admission in patients presenting to the emergency department with abdominal pain

**DOI:** 10.1186/s40001-023-01256-7

**Published:** 2023-08-17

**Authors:** Li-Tsung Lin, Sheng-Feng Lin, Chun-Chieh Chao, Hui-An Lin

**Affiliations:** 1 School of Medicine, College of Medicine, Taipei Medical University, Taipei, Taiwan; 2 Johns Hopkins Bloomberg School of Public Health, Johns Hopkins University, 501 St Paul St, Baltimore, MD 21202 USA; 3 Department of Medical Education, Taipei Veterans General Hospital, Taipei, Taiwan; 4 Department of Public Health, School of Medicine, College of Medicine, Taipei Medical University, Taipei, Taiwan; 5 School of Public Health, College of Public Health, Taipei Medical University, Taipei, Taiwan; 6 Department of Emergency Medicine, Taipei Medical University Hospital, No. 250, Wuxing St, Xinyi District, Taipei, 110 Taiwan; 7 Department of Emergency Medicine, School of Medicine, College of Medicine, Taipei Medical University Hospital, Taipei, Taiwan; 8 Graduate Institute of Clinical Medicine, School of Medicine, College of Medicine, Taipei Medical University, Taipei, Taiwan; 9 Graduate Institute of Public Health, College of Public Health, Taipei Medical University, No. 252, Wuxing St, Xinyi District, Taipei, 110 Taiwan

**Keywords:** Abdominal pain, Emergency department, Return visit

## Abstract

**Background:**

Unscheduled return visits (URVs) to the emergency department (ED) constitute a crucial indicator of patient care quality.

**Objective:**

We aimed to analyze the clinical characteristics of patients who visited the ED with abdominal pain and to identify the risk of URVs with admission (URVAs) from URVs without admission (URVNAs).

**Methods:**

This retrospective study included adult patients who visited the ED of Taipei Medical University Hospital because of abdominal pain and revisited in 72 h over a 5-year period (January 1, 2014, to December 31, 2018). Multivariable logistic regression analysis was employed to identify risk factors for URVAs and receiver operating characteristic (ROC) curve analysis was performed to determine the efficacy of variables predicting URVAs and the optimal cut-off points for the variables. In addition, a classification and regression tree (CART)-based scoring system was used for predicting risk of URVA.

**Results:**

Of 702 eligible patients with URVs related to abdominal pain, 249 had URVAs (35.5%). In multivariable analysis, risk factors for URVAs during the index visit included execution of laboratory tests (yes vs no: adjusted odds ratio [AOR], 4.32; 95% CI 2.99–6.23), older age (≥ 40 vs < 40 years: AOR, 2.10; 95% CI 1.10–1.34), Level 1–2 triage scores (Levels 1–2 vs Levels 3–5: AOR, 2.30; 95% CI 1.26–4.19), and use of ≥ 2 analgesics (≥ 2 vs < 2: AOR, 2.90; 95% CI 1.58–5.30). ROC curve analysis results revealed the combination of these 4 above variables resulted in acceptable performance (area under curve: 0.716). The above 4 variables were used in the CART model to evaluate URVA propensity.

**Conclusions:**

Elder patients with abdominal pain who needed laboratory workup, had Level 1–2 triage scores, and received ≥ 2 doses of analgesics during their index visits to the ED had higher risk of URVAs.

**Supplementary Information:**

The online version contains supplementary material available at 10.1186/s40001-023-01256-7.

## Introduction

An unscheduled return visit (URV) to the emergency department (ED) can be defined as an unexpected return to the ED after the index visit with similar complaints. URVs constitute an important indicator for monitoring patient care and medical performance [1]. A high URV rate may indicate insufficient initial medical care [1–5]. The incidence of URVs is estimated between 9 and 48%, and some URVs have been considered to be preventable [6, 7]. URVs to the ED within 72 h is used as an indicator for evaluating quality of care [1–5]; however, the efficacy of this URV-based metric has been questioned [8]. Distinguishing between “URVs with admission (URVAs)” and “URVs with no admission (URVNAs)” adds an additional level of accuracy in the assessment of quality of care in the ED [9, 10].

Since abdominal pain is a leading cause of ED visits [11], and also the main symptom in various physical conditions, emergency physicians often face challenges in determining the predisposition at short notice and with limited clinical clues. Moreover, for ED revisits, abdominal pain is the most common chief complaint as well [12–16]. Crystal et al. demonstrated that abdominal pain accounted for the highest proportion of URVs (22.2%), followed by fever (21.0%) and other gastrointestinal symptoms (19.7%) [17]. To stratify the risk of abdominal pain is difficult because even symptoms and signs of acute abdomen could be atypical and nonspecific in the initial phase [5, 18]. Despite improvements in knowledge and techniques over time, there was a trivial decrease of the URV rate in emergency medical care. Kuan investigated the URV rate over a 9-year period and revealed that the URV rate only declined from 25.1 to 22.2% [17]. Hence, abdominal-pain-related URVs remain a leading challenge in the ED setting.

Studies have analyzed the characteristics of patients with URVs to the ED [2, 5, 10, 19]; nevertheless, according to our review of the literature, no study has focused on the relationship between URVs and specific symptoms or diseases. Accordingly, to fill this literature gap, we included patients who revisited the ED because of abdominal pain in this study. To reduce URV rates, we aimed to investigate the relevant risk factors for URVAs.

## Methods

### Data source

We extracted the patient profiles from the administrative database of the Emergency Department of Taipei Medical University Hospital (TMUH-ED), which is a university hospital and level I trauma center. The TMUH-ED database preserves copies of all patients’ medical records in electronic form. This study was approved by the Joint Institutional Review Board of Taipei Medical University (approval number: N202103017). Informed consent was waived because all records were either anonymous or de-identified.

### Patient selection

We consecutively enrolled patients aged ≥ 20 years who visited the ED and then revisited the ED within 72 h of the index visit during the period between January 1, 2014, and December 31, 2018. To identify a subgroup of patients with URVs related to abdominal pain, we first excluded patients whose diagnoses based on International Classification of Diseases, Tenth Revision (ICD-10) codes during the revisit were not related to abdominal pain. Second, we excluded patients with pelvic diseases such as pelvic inflammatory disease or pregnancy-related problems during the revisit. Subsequently, abdominal-pain-related symptoms and diagnoses made on the basis of ICD-10 codes during the index visit were confirmed by Hui-An Lin and Li-Tsung Lin. Lastly, we excluded patients who were discharged against medical advice or those who had been transferred to the ED from other departments during either the initial or second visits.

### Variable selection

The selected variables were broadly classified into 2 categories: patient-related and system-related variables. The patient-related variables comprised sex, age (20–34, 35–49, 50–64, ≥ 65 year), triage score (Levels 1–5), vital signs (body temperature and pulse rate), pain score (based on numeric rating system: 0–3, 4–7, and 8–10), blood pressure (systolic blood pressure [SBP] and diastolic blood pressure [DBP]), tenderness or rebound tenderness, potentially immunocompromised status (cancer, diabetes mellitus, chronic kidney disease), or history of abdominal surgery. The system-related variables comprised duration of ED stay (0–2, ≥ 2 h), number of analgesics used, performance of initial examination (order of laboratory tests and imaging procedures), and history of TMUH visits.

The Taiwan Triage and Acuity Scale [20], which is widely used at hospitals in Taiwan, classifies patients in the ED into 5 levels on the basis of the acuity of illness: Level 1 (resuscitation), Level 2 (emergent), Level 3 (urgent), Level 4 (less urgent), and Level 5 (not urgent). Most patients with abdominal pain were triaged as a level 3; however, the patients who had associated symptoms and signs of severe pain (pain score of 8–10), SBP of < 90 mmHg, pulse rate of > 140 or < 50 beat/min, and fever up to 39 ℃ were escalated to the higher levels of 1 or 2. Accordingly, patients with abdominal pain were categorized into two groups (levels of 1–2 and 3–5, respectively). In this study, we considered the number of analgesics used, regardless of the route of administration, as an indicator of the need for pain control. We also included the decision for laboratory examination or imaging surveys—made by emergency physicians on the basis of their clinical judgments—as an independent factor in our study. These levels, along with system-related variables, were considered to indicate the severity of illness and degree of pain control required for the patients in our study cohort.

### Statistical analysis

We compared URVA and URVNA groups by applying the Chi-square test for categorical variables and the Mann–Whitney *U* test for continuous variables. Thereafter, univariate and multivariate logistic regression analyses were performed to obtain odds ratios and to identify the significant variables. In the multivariate analysis, we adopted two strategies for the explanatory variables: (1) the first strategy was by putting all clinical meaningful variables which showed statistically significant in univariable analysis (in our models 1 and 2). (2) the second strategy was by selecting the variables through backward elimination (in our models 3 and 4). In addition, the Hosmer–Lemeshow statistics were used to examine the goodness of fit for logistic regression models, and the Akaike information criterion (AIC) was used to determine which model had the best fit for the data. The model with the lowest value of AIC was the most preferred model. We used receiver operating characteristic (ROC) curves to examine the correlation between the identified significant variables and URVAs. The Youden’s index (sensitivity + specificity—1) was used to obtain the optimal cut-off value for age. As a validation analysis, a classification and regression tree (CART)—a predictive model that applies a dichotomous decision process and splits data according to a certain cut-off value—was used to establish clinical guidelines for identifying URVAs. In the CART model, all significant variables in the logistic regression analysis were included and were determined which stratification to conduct at every stage. All statistical analyses were performed using R software 4.1 (R Core Team, 2021) and SAS 9.4 (SAS Institute, Cary, NC, USA). The statistical significance was defined as *p* < 0.05.

## Results

### Study population

**Fig. 1:**
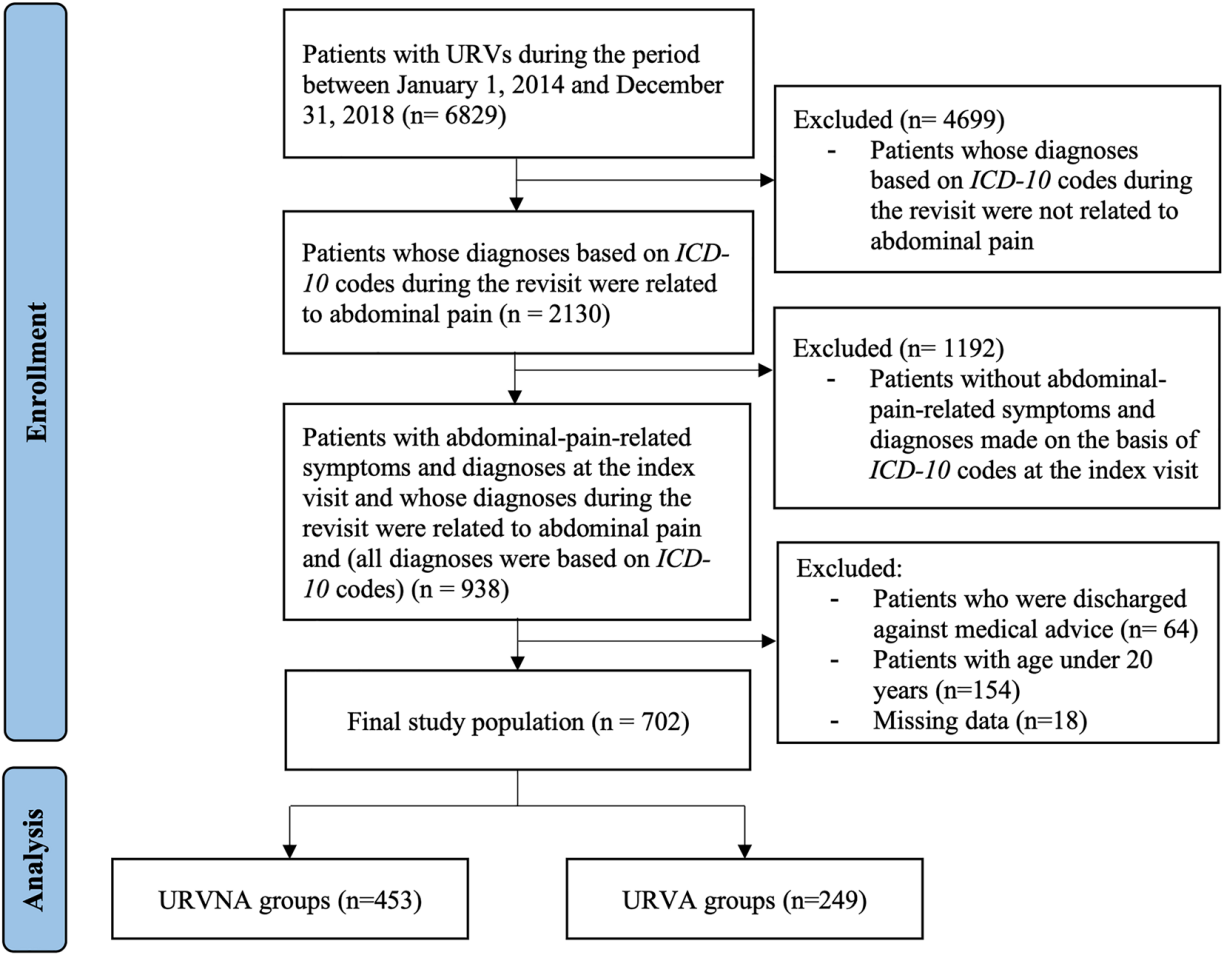
Patient enrollment and data analysis procedures

During the 5-year study period, a total of 6829 patients with URVs were identified in our database; of these patients, 702 met the study inclusion criteria. Among the 702 patients with URVs, 249 (35.5%) and 453 (64.5%) had URVAs and URVNAs, respectively. The flowchart of the study procedures, including the patient enrollment and outcome analysis procedures is displayed in Fig. 1. The detailed information of included ICD-10 codes included in present study is demonstrated in Additional file 1: Table S1.

### Descriptive analysis

The characteristics of the study population are shown in Table 1. The median age of the 702 patients included in this study was 47 years (in a range of 20 to 103 years), and 42.9% of the included patients were men. The majority of the patients’ visits were not urgent (triage Levels 3–5; 93.4%). The laboratory test and imaging execution rates were 61.0% and 58.4%, respectively. A comparison of the URVA and URVNA groups revealed that the URVA group was older (median age: 52 vs 42 year; *P* < 0.001), had a greater history of prior hospital visits (94.0% vs 87.9%; *P* = 0.010), had a longer ED duration (median hours: 2.2 vs 1.5 h; *P* = 0.001), had higher triage scores (Levels 1–2: 10.0% vs 4.6%; *P* = 0.006), had higher laboratory test execution rates (81.1% vs 49.9%; *P* < 0.001), had higher imaging execution rates (71.5% vs 51.2%; *P* < 0.001), and was prescribed more analgesics (≥ 2: 11.2% vs 4.2%; *P* < 0.001). Moreover, a comparison of the URVA and URVNA groups indicated that the URVA group exhibited lower SBP levels (SBP ≥ 130 mmHg: 41.0% vs 53.2%; *P* = 0.002) and more comorbidities (history of cancer: 16.1% vs 8.4%, *P* = 0.002; history of abdominal surgery: 30.5% vs 19.9%, *P* = 0.001; and history of diabetes mellitus: 15.3% vs 7.9%, *P* = 0.003). The 2 groups did not differ significantly in terms of other variables, namely gender, body temperature, heart rate, DBP, pain score, chronic kidney disease, tenderness (including rebound tenderness), or sonography performance.

**Table 1:** Comparisons of characteristics in URVNA and URVA populations

Variables	Total (*N* = 702)	Disposition		*P* value
		URVNA (*n* = 453)	URVA (*n* = 249)	
Age (year) [IQR]	47 [32–64]	42 [30–59]	52 [39–68]	< 0.001*
Age group (*N*, %)				< 0.001*
20–34	208 (29.6)	167 (36.9)	41 (16.5)	
35–49	171 (24.4)	98 (21.6)	73 (29.3)	
50–65	156 (22.2)	95 (21.0)	61 (24.5)	
> 65	167 (23.8)	93 (20.5)	74 (59.2)	
Gender (male, %)	301 (42.9)	194 (42.8)	107 (43.0)	0.970
Number of ED visits in a year (*N*) [IQR]	2 [1–3]	2 [1–3]	2 [1–3]	0.552
Number of ED visits in a year (*N*, %)				0.027*
0–4	588 (83.8)	376 (83.0)	212 (85.1)	
4–8	54 (7.7)	31 (6.8)	23(9.2)	
8–12	23 (3.3)	14 (3.1)	9 (3.6)	
> 12	37 (5.3)	32 (7.1)	5 (2.0)	
ED duration (h) [IQR]	1.7 [1.0–2.7]	1.5 [0.7–2.4]	2.2 [1.5–3.4]	0.001*
ED duration (*N*, %)				< 0.001*
< 2 h	407 (58.0)	302 (66.7)	105 (42.2)	
≥ 2 h	295 (42.0)	151 (33.3)	144 (57.8)	
Triage score (*N*, %)				0.006*
Levels 1–2	46 (6.6)	21 (4.6)	25 (10.0)	
Levels 3–5	656 (93.4)	432 (95.4)	224 (90.0)	
Vital signs at the ED				
Body temperature (℃)	36.6 [36.3–36.9]	36.7 [36.4–36.9]	36.6 [36.3–36.9]	0.229
Body temperature (*N*, %)				0.595
< 37.5 ℃	652 (92.9)	419 (92.5)	233 (93.6)	
≥ 37.5 ℃	50 (7.1)	34 (7.5)	16 (6.4)	
Heart rate				0.335
60–100 bpm	538 (76.6)	342 (75.7)	196 (78.7)	
< 60 or > 100 bpm	164 (23.4)	111 (24.5)	53 (21.3)	
Blood pressure				
SBP (*N*, %)				0.002*
≥ 130 mmHg	343 (48.9)	241 (53.2)	102 (41.0)	
< 130 mmHg	359 (51.1)	212 (46.8)	147 (59.0)	
DBP (*N*, %)				0.067
≥ 80 mmHg	345 (49.1)	242 (53.4)	115 (46.2)	
< 80 mmHg	357 (50.9)	211 (46.6)	134 (53.8)	
Pain score (NRS, *N*, %)				0.938
0–3	180 (25.6)	118 (26.0)	62 (24.9)	
4–7	496 (70.7)	318 (70.2)	178 (71.5)	
8–10	26 (3.7)	17 (3.8)	9 (3.6)	
History of previous hospital visit (*N*, %)	70 (10.0)	398 (87.9)	234 (94.0)	0.010*
Medical history				
History of cancer (*N*, %)	78 (11.1)	38 (8.4)	40 (16.1)	0.002*
History of abdominal surgery (*N*, %)	166 (23.6)	90 (19.9)	76 (30.5)	0.001*
DM (*N*, %)	74 (10.5)	36 (7.9)	38 (15.3)	0.003*
CKD (*N*, %)	16 (2.1)	8 (1.8)	7 (2.8)	0.360
Physical examination of abdomen				
Tenderness/rebound tenderness (*N*, %)	294 (41.9)	182 (40.2)	112 (45.0)	0.217
Rebound tenderness (*N*, %)	20 (2.8)	10 (2.2)	10 (4.0)	0.168
Workup at the ED				
Performance of laboratory test (*N*, %)	428 (61.0)	226 (49.9)	202 (81.1)	< 0.001*
Performance of image (*N*, %)	410 (58.4)	232 (51.2)	178 (71.5)	< 0.001*
Performance of sonography (*N*, %)	152 (21.7)	97 (21.4)	55 (22.1)	0.835
Number of analgesics used§ (*N*, %)	47 (6.7)	19 (4.2)	28 (11.2)	< 0.001*

*URVNA* unscheduled return visit without admission, *URVA* unscheduled return visit with admission, *N* number, *ED* emergency department, *bpm* beats per minute, *SBP* systolic blood pressure, *DBP* diastolic blood pressure, *NRS* numeric rating scale, *DM* diabetes mellitus, *CKD* chronic kidney disease

^§^Number of analgesics used: 2 or more analgesics were prescribed for pain control, inclusive of oxethazaine, acetaminophen, ketorolac, ibuprofen, and NSAIDs

Categorical data are presented as numbers (%); continuous data are expressed as median [IQR]

^*^ Statistical significance (*α* = 0.05)

### Predictors of URVAs

The results of comparative analyses between the URVA and URVNA groups are presented in Table 2. In our logistic regression analysis, age was employed as the continuous variable (models 1 and 3) and categorical variable (models 2 and 4). To obtain the best cut-off value for age, we found the age at ≥ 40 vs < 40 year had the highest Youden’s index (Additional file 1: Table S2). All models attained the criteria of stable goodness of fit in the Hosmer–Lemeshow test. For comparison of models, the second strategy of backward elimination was more favored than the first strategy due to the lower AIC values. Moreover, the model 4 with age as the categorical variable was selected as the best model due to the lowest AIC value of 55.17.

**Table 2:** Predictors of URVA in 702 patients: univariable and multivariable analysis results

Characteristics	Univariable analysis	*P* value	Multivariable analysis (^a^ Model 1)	*P* value	Multivariable analysis (^a^ Model 2)	*P* value	Multivariable analysis (^b^ Model 3)	*P* value	Multivariable analysis (^b^ Model 4)	*P* value
	OR (95% CI)		OR (95% CI)		OR (95% CI)		OR (95% CI)		OR (95% CI)	
Age (per 10-year increase)	1.23 (1.10–1.34)	< 0.001*	1.08 (0.97–1.20)	0.143			1.18 (1.10–1.34)	< .001*		
Age (≥ 40 vs < 40 year)	2.48 (1.76–3.48)	< 0.001*			1.56 (1.04–2.33)	0.031*			2.10 (1.47–2.99)	< .001*
ED duration (≥ 2 vs < 2 h)	2.74 (2.00–3.77)	< 0.001*	1.24 (0.84–1.83)	0.283	1.26 (0.85–1.86)	0.248				
Triage score (levels 1–2 vs levels 3–5)	2.30 (1.26–4.19)	0.007*	1.82 (0.97–3.42)	0.080	1.76 (0.94–3.32)	0.080	1.88 (1.01–3.52)	0.049*	1.78 (0.95–3.34)	0.074
SBP (≥ 130 vs < 130 mmHg)	1.64 (1.20–2.24)	0.002*	1.31 (0.92–1.86)	0.131	1.30 (0.92–1.84)	0.140				
History of previous hospital visit (yes vs no)	2.16 (1.19–3.90)	0.011*	1.50 (0.79–2.84)	0.212	1.47 (0.78–2.80)	0.235				
History of cancer (yes vs no)	2.09 (1.30–3.36)	0.002*	1.41 (0.84–2.52)	0.183	1.41 (0.82–2.43)	0.220				
History of abdominal surgery (yes vs no)	1.77 (1.24–2.53)	0.002*	1.23 (0.82–1.84)	0.326	1.21 (0.81–1.81)	0.357				
DM (yes vs no)	2.09 (1.29–3.39)	0.003*	1.50 (0.87–2.57)	0.141	1.44 (0.84–2.46)	0.182				
Execution of laboratory tests (yes vs no)	4.32 (2.99–6.23)	< 0.001*	2.92 (1.87–4.56)	< 0.001*	2.90 (1.86–4.53)	< 0.001*	3.69 (2.53–5.37)	< 0.001*	3.70 (2.54–5.39)	< 0.001*
Execution of imaging analysis (yes vs no)	2.39 (1.72–3.33)	< 0.001*	1.24 (0.84–1.81)	0.282	1.22 (0.83–1.79)	0.310				
Number of analgesics used (≥ 2 vs < 2)	2.90 (1.58–5.30)	0.001*	1.93 (1.01–3.72)	0.048*	1.91 (1.01–3.72)	0.052	2.10 (1.12–3.95)	0.022*	2.06 (1.09–3.87)	0.025*
AIC			774.64		386.84		455.60		55.17	
Hosmer–Lemeshow test				0.359		0.397		0.272		0.626

*OR* odds ratio, *ED* emergency department, *SBP* systolic blood pressure, *DM* diabetes mellitus, *AIC* Akaike information criterion, *CI* confidence interval

^a^Model 1 and ^a^Model 2 included all the statistically and clinically significant variables; ^b^Model 3 and ^b^

Model 4 included variables through backward elimination. Age was used as a continuous variable (models 1 and 3) and a categorical variable (models 2 and 4)

^*^Statistical significance was defined as *p* < 0.05

In model 4, older age (≥ 40 vs < 40 year: AOR, 2.10; 95% CI 1.47–2.99), execution of laboratory tests (yes vs no: AOR, 3.70; 95% CI 2.54–5.39), and administration of multiple analgesics (≥ 2 vs < 2: AOR, 2.06; 95% CI 1.09–3.87) were associated with increased odds of URVA. Both models 3 and 4 revealed consistent results.

### Correlation of predictors with URVAs

**Fig. 2:**
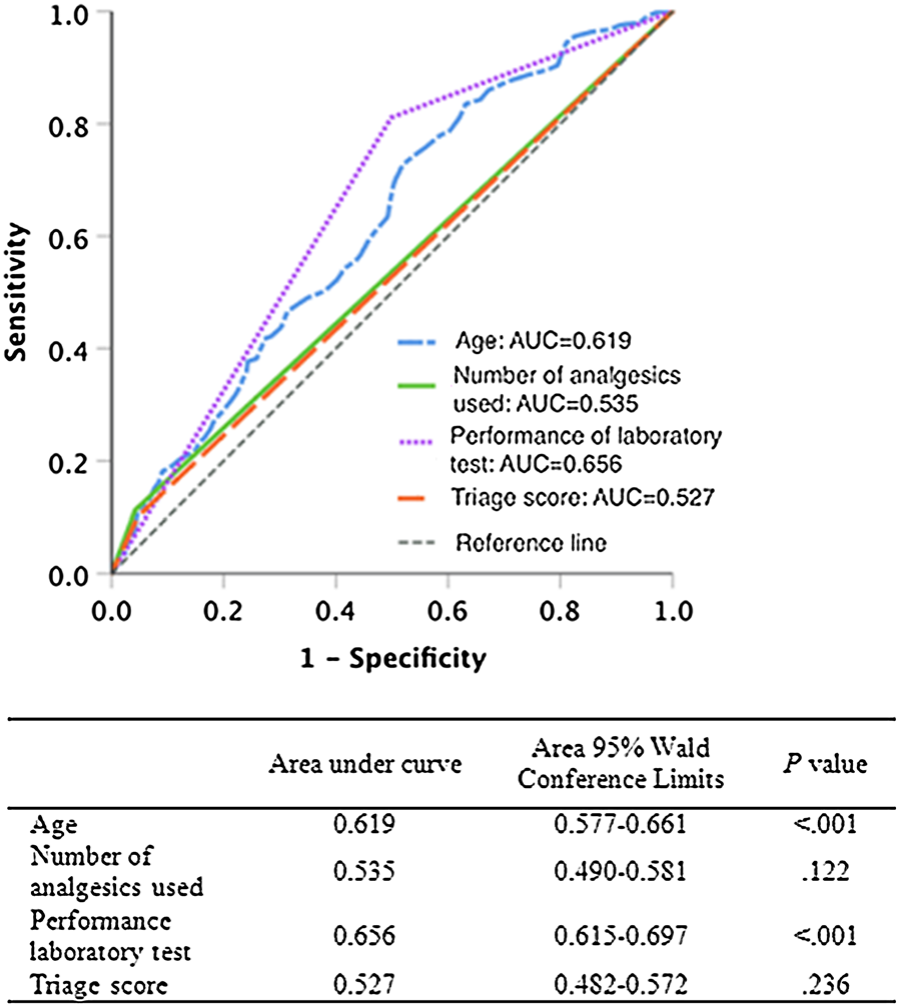
Receiver of operating characteristic (ROC) curve analysis results for the variables predicting URVA risk. Given is the ROC curve for the association between the variables and URVAs in all patients who met the inclusion criteria. The variables were age, number of analgesics used, execution of laboratory tests, and triage score. The reference line reveals no difference from chance. *AUC* area under curve

The ROC curves of risk factors for URVA are displayed in Fig. 2. The areas under the curves (AUCs) for triage score, administration of multiple analgesics, age (serving as a continuous variable), and execution of laboratory tests were 0.527 (*P* = 0.236), 0.535 (*P* = 0.122), 0.619 (*P* < 0.001), and 0.656 (*P* < 0.001), respectively. Through these 4 risk factors, the model could achieve acceptable discrimination performance (AUC = 0.716; *P* < 0.001) in the prediction of URVAs.

### Construction of new scoring system for predicting URVAs

**Fig. 3:**
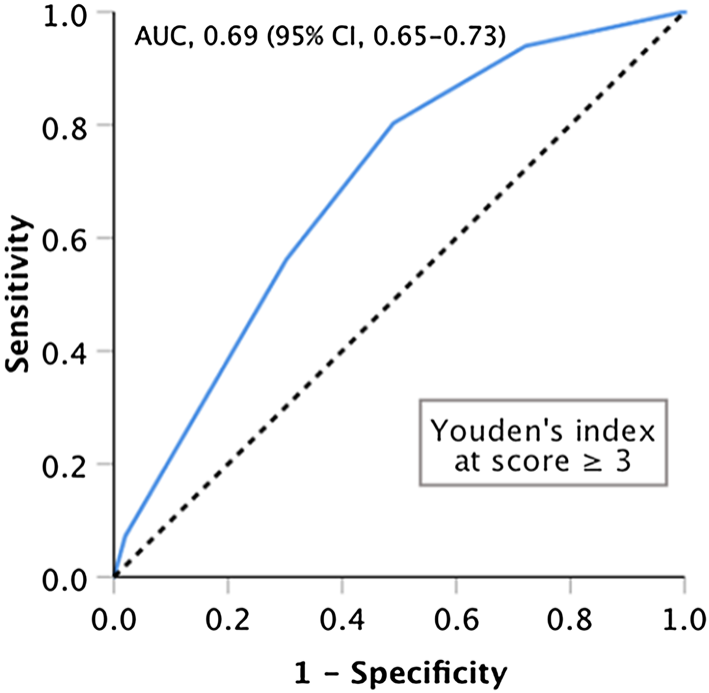
Receiver of operating characteristic (ROC) curve analysis results for the established scoring system for predicting URVAs. Given is the ROC curve for the association between the new scoring system and URVAs in all patients who met the inclusion criteria. The dashed line represents a reference line revealing no difference from chance. *AUC* area under the curve

Our logistic regression analysis results indicated that age ( ≥ 40 year), triage score (Levels 1–2), execution of laboratory tests, and number of analgesics used (≥ 2) were significant variables predicting URVAs after the index visit to the ED (Table 3). Cutoff points for these variables were derived by using the maximum value of the Youden's index on the corresponding ROC curves (Additional file 2: Fig. S1). Thus, these variables were used to establish a new scoring system for predicting URVAs. A score of 1 was assigned to age (≥ 40 year), triage score (Levels 1–2), and number of analgesics used (≥ 2), and a score of 2 was assigned to execution of laboratory tests; thus, the total score for the system ranged from 0 to 5. On the basis of the maximum value of Youden's index on ROC curves, the optimal cut-off point for the variables predicting URVAs was 3, with the corresponding sensitivity and specificity being 63.1% and 68.0%, respectively (Fig. 3). Accordingly, the established system achieved acceptable discrimination performance in predicting URVAs (AUC = 0.709; 95% CI 0.672–0.746) and acceptable calibration (Hosmer–Lemeshow test; *P* = 0.483).

**Table 3:** New scoring system for predicting URVAs

Characteristics	Multivariable analysis		Points assigned	Sensitivity (95% CI)	Specificity (95% CI)
	OR (95% CI)	*P* value			
Adopted model for building new scoring system					
Age (≥ 40 vs < 40 year)	2.12 (1.49–3.00)	< 0.001*	1		
Triage (levels 1–2 vs levels 3–5)	1.75 (0.93–3.28)	0.0834	1		
Execution of laboratory tests (yes vs no)	3.74 (2.57–5.45)	< 0.001*	2		
Number of analgesics used (≥ 2 vs < 2)	2.02 (1.08–3.81)	0.029*	1		
Area under curve	0.719 (0.680–0.757)				
Hosmer–Lemeshow test	1.55 (9 groups)	0.9805			
New scoring system (per score increase)	1.96 (1.69–2.27)	< 0.001*			
Score of ≥ 1	6.69 (3.69–12.14)	< 0.001*		94.8% (92.0–97.5%)	26.9% (22.9–31.0%)
Score of ≥ 2	4.32 (2.97–6.28)	< 0.001*		82.3% (92.0–97.5%)	48.1% (43.5–52.7%)
Score of ≥ 3	3.63 (2.62–5.01)	< 0.001*		63.1% (92.0–97.5%)	68.0% (63.7–72.3%)
Score of ≥ 4	4.92 (2.68–9.02)	< 0.001*		15.3% (10.8–19.7%)	96.5% (94.8–98.2%)
Area under curve	0.709 (0.672–0.746)				
Hosmer–Lemeshow test	2.46 (5 groups)	0.4832			

*CI* confidence interval, *OR* odds ratio, *URVA* unscheduled return visit with admission

^*^Statistical significance (*α* = 0.05)

### CART analysis

**Fig. 4:**
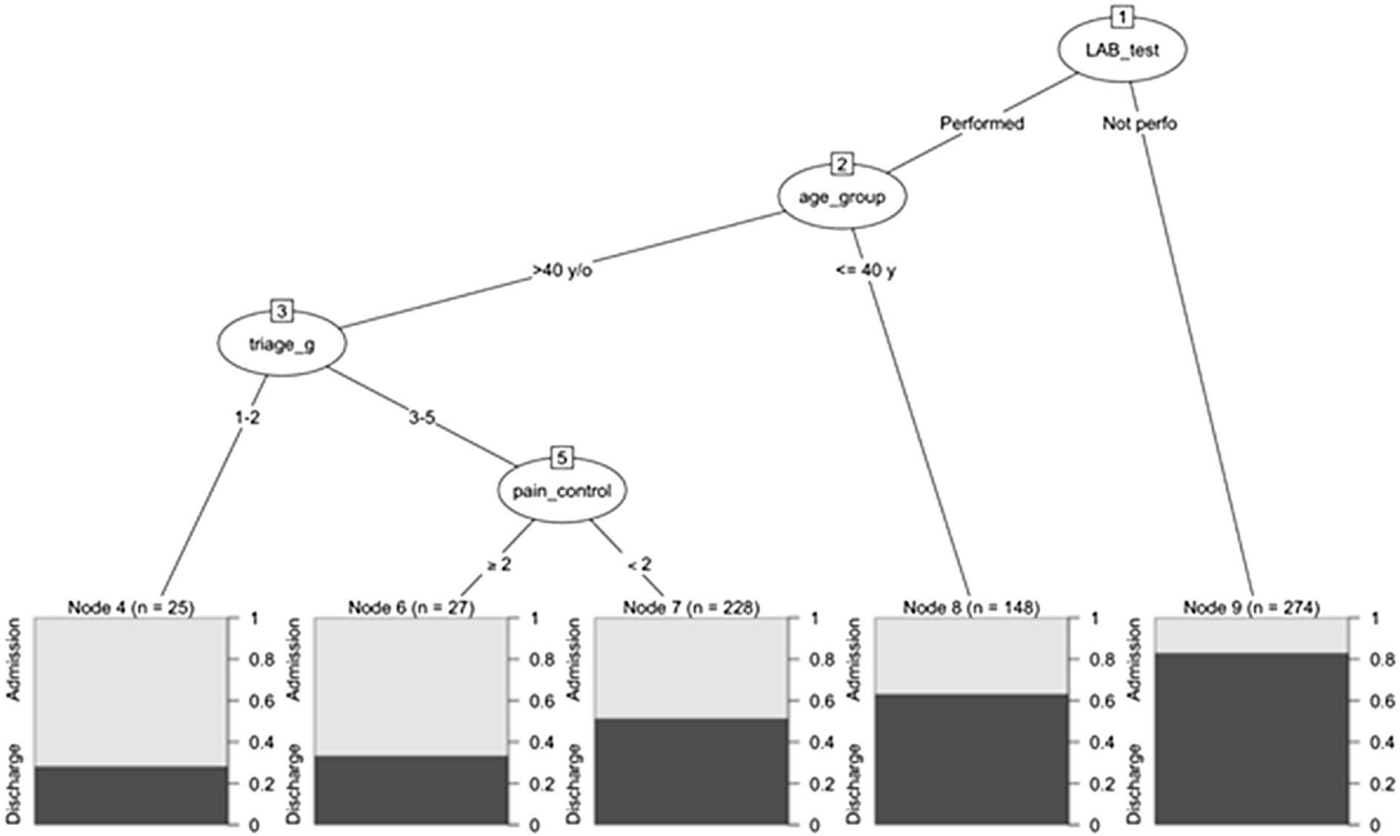
Four predictive variables were analyzed using decision tree analysis through R software 4.1 (R foundation). URVAs were predicted by execution of laboratory tests, age, triage score, and number of analgesics used

To explore influential predictors of URVAs, a CART model was established to predict URVAs and URVNAs (Fig. 4). The 4 variables included in this model were execution of laboratory tests (yes vs no), age (≥ 40 vs < 40 year), triage score (Levels 1–2 vs Levels 3–5), and number of analgesics administered (≥ 2 vs < 2). Execution of laboratory tests was the first determining predictor in this model. Among 274 patients who did not undergo laboratory tests, only 47 (17%) had URVAs. Of 428 patients who underwent laboratory tests, age (≥ 40 vs < 40 year) was a predictor of URVAs. Patients aged < 40 years (*n* = 148) were less likely to have URVAs (*n* = 55; 37.2%) compared with other patients. Among patients aged > 40 years, those with a triage score of Levels 1–2 (*n* = 25) were more likely to have URVAs (*n* = 18; 72.0%) compared with other patients. Among patients with a triage score of Levels 3–5 (*n* = 255), number of analgesics administered was a predictor of subsequent branching. Patients who received 2 or more analgesics (*n* = 27) were more likely to have URVAs (*n* = 18; 66.7%) compared with other patients.

## Discussion

In this study, we constructed a new scoring system by employing several significant risk factors for URVAs related to abdominal pain. The factors considered in this system are outlined as follows: age (≥ 40 year), triage score (Levels 1–2), execution of laboratory tests, and number of analgesics used (≥ 2). We observed that a score of ≥ 3 in this system indicated a higher risk of URVAs.

According to our CART analysis, whether patients had laboratory workup was most related to their URVAs. Similar to our result, Liu and colleagues demonstrated a positive correlation between execution of laboratory tests and readmission within 72 h after discharge from the ED [19]. Consideration of the need of laboratory workup is complicated to physicians and should be based on comprehensive integration of characteristics of patients, clinical presentation, and physical examination finding. Although laboratory tests alone are insufficient for diagnosing acute abdomen [21], combining them with history-taking and physical examination can provide clinicians with objective information to identify physical conditions and narrow down the differential diagnosis [22, 23]. Moreover, the need for laboratory tests indicates the complexity and uncertainty of a patient’s presentation at the ED. Early study had revealed that several non-medical factors, including need of laboratory workup, were concerned by physicians in their decision-making about inpatient care [24]. Therefore, physicians are more likely to execute laboratory tests under the impression of more complex abdominal pain. On the other hand, whether performing image workup is not strongly associated with the revisit in our result. An earlier study which explored the trend of using imaging studies to evaluate abdominal pain in ED indicated that increasing use of CT or sonography did not influence the rate of detection of appendicitis, gallbladder disease and diverticulitis [25]. The value of abdominal CT imaging was to help confirm the diagnosis after comprehensive clinical evaluation. Otherwise, it was no additional benefit for routine use of CT on evaluating abdominal pain.

To comply with the National Health Insurance (NHI) System in Taiwan, the physicians must prescribe laboratory and image workup with proper indications. Medical expenses would only be paid by the NHI if they fulfilled the documented clinical indications. Otherwise, the responsibility fell upon medical providers. We considered the reasons for higher use of laboratory tests than imaging studies as follows. In general, imaging studies were performed to confirm the diagnosis, such as ileus, appendicitis, ischemic bowel, etc., rather than an indispensable tool for most clinical conditions after comprehensive history-taking, physical examination, and laboratory tests. In addition, laboratory tests were prescribed in emergency departments for multiple clinical purposes. While these tests assisted in surveying the cause of abdominal pain only, they helped evaluate the complications in other organ systems, such as acute kidney injury, electrolyte imbalance, anemia, etc. A common example was that the creatinine and pregnancy tests were prescribed for a young woman with abdominal pain.

Aging is another important risk for revisit. Evaluation of older adult patients in the ED is challenging because of their comorbidities, obscured clinical presentations, and fragile physical conditions [21, 26–29]. Older adults tend to be underserved by triage systems and experience higher ED mortality rates [30, 31]. Therefore, advanced age in many cases is an indicator to URVAs within 72 h [2, 10, 32–36]. However, evidence for this relationship is inconsistent in the literature. For example, Liu et al. determined no significant correlation between age and URVAs [19]; nevertheless, the mean age of the patients in their cohort was 46.8 years, which is younger than that reported in the aforementioned studies [2, 10, 32–36] and may have resulted in the underrepresentation of the influence of aging on URVAs.

Another noteworthy parameter in our study was the triage score, which was used to assess how imperative a patient needs to be managed. Triage consists of 5 acuity levels that indicate severity and enable paramedical staff to effectively prioritize patients [33]. Patients designated triage Level 1 or Level 2 usually have higher risks of intensive care unit admission, emergency surgery, and in-hospital cardiac arrest during an ED revisit [37]. Because older patients with abdominal pain are prone to being undertriaged [38], the combination of older age and higher triage scores (low acuity of triage) could increase their revisit rates and result in poor prognosis.

Although the degree of pain is subjective, scholars and clinicians had widely supported the importance of pain evaluation and considered pain as the fifth vital sign [39–43]. Appropriate pain control after systematic assessment potentially reduces the revisit rate in hospital care [44–46]. A patient’s request for analgesics for abdominal pain reflects their subjective impression of the severity of their illness. Appropriate pain control does not mask illness; instead, it relieves stress and allows patients to cooperate with medical staff during subsequent diagnostic tests [47, 48]. In our study, patients who requested painkillers more than once were prone to URVAs; therefore, ED physicians should be aware of the risk of URVAs that accompanies requests for additional analgesics.

According to our review of the literature, this is the first study to reveal the association between abdominal pain and the risk of URVAs. However, this study has several limitations. First, all data were extracted from a single database of a tertiary teaching hospital, and patients who visited a different hospital within 72 h of the index visit would have been overlooked by this study. Second, indications for admission at the revisit varied between physicians; detailed parameters must be used to conduct a comprehensive analysis of indications for admission. Third, because our study involved a retrospective design, we could not determine the causality of URVAs and its predictors. Therefore, additional prospective or randomized controlled trials should be conducted to confirm this association.

In conclusion, our study demonstrated that patients who underwent laboratory tests, were aged ≥ 40 years, had high triage scores, and received multiple analgesics during their index visit to the ED because of abdominal pain were at a relatively high risk of URVAs. Accordingly, on the basis of age (≥ 40 year), triage score (Levels 1–2), execution of laboratory tests, and number of analgesics used (≥ 2), we established a scoring system for screening patients with abdominal pain prior to ED discharge. In this system, a score of > 3 points should alert clinicians to consider additional risks for disease progression or deterioration in patients and the possible need for further inpatient treatment.

### Supplementary Information

**Additional file 1:** ICD-10 diagnosis codes and the Youden's index.

**Additional file 2: ** Optimal cut-off point.

## Data Availability

The data are not publicly available. With legal restrictions imposed by the government of Taiwan on the distribution of the personal health data in relation to the “Personal Information Protection Act”, requests for data need a formal proposal which should be directed to Joint Institutional Review Board of Taipei Medical University and Office Human Research, Taipei Medical University (tmujirb@gmail.com).
