# Supplementary material for: Predictors of 72-h unscheduled return visits with admission in patients presenting to the emergency department with abdominal pain
Source: Eur J Med Res. 2023 Aug 17;28:288. doi: 10.1186/s40001-023-01256-7 (PMC10433659; doi:10.1186/s40001-023-01256-7)
Supplement: Supplementary file 1 — Additional file 1: ICD-10 diagnosis codes and the Youden's index. [file 40001_2023_1256_MOESM1_ESM.docx]

Supplemental Table 1. ICD-10 diagnosis codes during the revisit of the study population.

| **ICD-10** | **Diagnosis** |
| --- | --- |
| **A09** | **Other gastroenteritis and colitis of infectious and unspecified origin** |
| **B18.8** | **Other chronic viral hepatitis** |
| **C16.9** | **Malignant neoplasm: Stomach, unspecified.** |
| **C18.0** | **Malignant neoplasm of cecum** |
| **C18.2** | **Malignant neoplasm: Ascending colon** |
| **C18.4** | **Malignant neoplasm: Transverse colon** |
| **C18.8** | **Malignant neoplasm of overlapping sites of colon** |
| **C18.9** | **Malignant neoplasm of colon, unspecified** |
| **C21.8** | **Malignant neoplasm: Overlapping lesion of rectum, anus and anal canal** |
| **C22.0** | **Liver cell carcinoma** |
| **C22.1** | **Intrahepatic bile duct carcinoma** |
| **C22.8** | **Malignant neoplasm of liver, primary, unspecified as to type** |
| **C24.9** | **Malignant neoplasm: Biliary tract, unspecified** |
| **C25.3** | **Malignant neoplasm of pancreatic duct** |
| **C25.9** | **Malignant neoplasm: Pancreas, unspecified** |
| **C48.1** | **Malignant neoplasm of specified parts of peritoneum** |
| **C78.6** | **Secondary malignant neoplasm of retroperitoneum and peritoneum** |
| **C78.7** | **Secondary malignant neoplasm of liver and intrahepatic bile ducts** |
| **D01.0** | **Carcinoma in situ of colon** |
| **D49.0** | **Neoplasm of unspecified behavior of digestive system** |
| **K21.0** | **Gastro-esophageal reflux disease with esophagitis** |
| **K21.9** | **Gastro-oesophageal reflux disease without oesophagitis** |
| **K25.3** | **Acute gastric ulcer without hemorrhage or perforation** |
| **K25.9** | **Gastric ulcer Unspecified as acute or chronic, without haemorrhage or perforation** |
| **K26.9** | **Duodenal ulcer Unspecified as acute or chronic, without haemorrhage or perforation** |
| **K27.0** | **Acute peptic ulcer, site unspecified, with hemorrhage** |
| **K27.9** | **Peptic ulcer, site unspecified Unspecified as acute or chronic, without haemorrhage or perforation** |
| **K28.3** | **Acute gastrojejunal ulcer without hemorrhage or perforation** |
| **K29.00** | **Acute gastritis without bleeding** |
| **K29.30** | **Chronic superficial gastritis without bleeding** |
| **K29.50** | **Unspecified chronic gastritis without bleeding** |
| **K29.70** | **Gastritis, unspecified, without bleeding** |
| **K29.71** | **Gastritis, unspecified, with bleeding** |
| **K29.81** | **Duodenitis with bleeding** |
| **K30** | **Functional dyspepsia** |
| **K31.89** | **Other diseases of stomach and duodenum** |
| **K31.9** | **Disease of stomach and duodenum, unspecified** |
| **K35.2** | **Acute appendicitis with generalized peritonitis** |
| **K35.3** | **Acute appendicitis with localized peritonitis** |
| **K35.80** | **Unspecified acute appendicitis** |
| **K37** | **Unspecified appendicitis** |
| **K40.20** | **Bilateral inguinal hernia, without obstruction or gangrene Not specified as recurrent hernia** |
| **K40.21** | **Bilateral inguinal hernia, without obstruction or gangrene, recurrent** |
| **K42.0** | **Umbilical hernia with obstruction, without gangrene** |
| **K52.9** | **Noninfective gastroenteritis and colitis, unspecified. colitis, diarrhoea, enteritis, gastroenteritis: infectious** |
| **K56.41** | **Fecal impaction** |
| **K56.5** | **Intestinal adhesions [bands] with obstruction (postinfection)** |
| **K56.60** | **Unspecified intestinal obstruction** |
| **K56.7** | **Ileus, unspecified** |
| **K57.12** | **Diverticulitis of small intestine without perforation, abscess or bleeding** |
| **K57.20** | **Diverticulosis of large intestine with perforation and abscess, without bleeding** |
| **K57.32** | **Diverticulitis of large intestine without perforation, abscess or bleeding** |
| **K59.00** | **Constipation, unspecified** |
| **K59.9** | **Functional intestinal disorder, unspecified** |
| **K63.1** | **Perforation of intestine (nontraumatic)** |
| **K65.0** | **Generalized (acute) peritonitis** |
| **K65.8** | **Other peritonitis** |
| **K65.9** | **Peritonitis, unspecified** |
| **K68.12** | **Psoas muscle abscess** |
| **K68.9** | **Other disorders of retroperitoneum** |
| **K70.31** | **Alcoholic cirrhosis of liver with ascites** |
| **K73.9** | **Chronic hepatitis, unspecified** |
| **K74.60** | **Unspecified cirrhosis of liver** |
| **K75.9** | **Inflammatory liver disease, unspecified** |
| **K76.9** | **Liver disease, unspecified** |
| **K80.00** | **Calculus of gallbladder with acute cholecystitis Without mention of obstruction of biliary tract** |
| **K80.01** | **Calculus of gallbladder with acute cholecystitis with obstruction** |
| **K80.12** | **Calculus of gallbladder with acute and chronic cholecystitis without obstruction** |
| **K80.20** | **Calculus of gallbladder without cholecystitis Without mention of obstruction of biliary tract** |
| **K80.21** | **Calculus of gallbladder without cholecystitis With obstruction of biliary tract** |
| **K80.40** | **Calculus of bile duct with cholecystitis Without mention of obstruction of biliary tract** |
| **K80.50** | **Calculus of bile duct without cholangitis or cholecystitis Without mention of obstruction of biliary tract** |
| **K80.60** | **Calculus of gallbladder and bile duct with cholecystitis, unspecified, without obstruction** |
| **K80.61** | **Calculus of gallbladder and bile duct with cholecystitis, unspecified, with obstruction** |
| **K80.70** | **Calculus of gallbladder and bile duct without cholecystitis without obstruction** |
| **K80.80** | **Other cholelithiasis Without mention of obstruction of biliary tract** |
| **K80.81** | **Other cholelithiasis with obstruction** |
| **K81.0** | **Acute cholecystitis** |
| **K81.2** | **Acute cholecystitis with chronic cholecystitis** |
| **K81.9** | **Cholecystitis, unspecified** |
| **K82.9** | **Disease of gallbladder, unspecified** |
| **K83.0** | **Diseases of the digestive system** |
| **K83.1** | **Obstruction of bile duct** |
| **K83.8** | **Other specified diseases of biliary tract** |
| **K85.0** | **Idiopathic acute pancreatitis** |
| **K85.2** | **Alcohol induced acute pancreatitis** |
| **K85.8** | **Other acute pancreatitis** |
| **K85.9** | **Acute pancreatitis, unspecified** |
| **K86.0** | **Alcohol-induced chronic pancreatitis** |
| **K86.1** | **Other chronic pancreatitis** |
| **K86.9** | **Disease of pancreas, unspecified** |
| **K91.3** | **Postprocedural intestinal obstruction** |
| **K92.1** | **Melena** |
| **K92.2** | **Gastrointestinal haemorrhage, unspecified** |
| **K92.81** | **Gastrointestinal mucositis (ulcerative)** |
| **R10.0** | **Acute abdomen** |
| **R10.10** | **Upper abdominal pain, unspecified** |
| **R10.11** | **Right upper quadrant pain** |
| **R10.12** | **Left upper quadrant pain** |
| **R10.13** | **Epigastric pain** |
| **R10.30** | **Lower abdominal pain, unspecified** |
| **R10.31** | **Right lower quadrant pain** |
| **R10.813** | **Right lower quadrant abdominal tenderness** |
| **R10.83** | **Colic** |
| **R10.84** | **Generalized abdominal pain** |
| **R10.9** | **Unspecified abdominal pain** |
| **R11.0** | **Nausea** |
| **R11.14** | **Bilious vomiting** |
| **R11.2** | **Nausea with vomiting, unspecified** |
| **R14.3** | **Flatulence** |
| **R17** | **Unspecified jaundice** |
| **R18.8** | **Other ascites** |
| **R19.5** | **Other fecal abnormalities** |
| **R19.7** | **Diarrhea, unspecified** |
| **R19.8** | **Other specified symptoms and signs involving the digestive system and abdomen** |

**Supplemental Table 2. Youden’s index by different cut-off on the ROC Curve**

| Test Result Variable(s): Age (continuous) | | | |
| --- | --- | --- | --- |
| Positive if Greater Than or Equal To | Sensitivity | 1 - Specificity | ^a^ Youden’s index |
| 19.00 | 1.000 | 1.000 | 0 |
| 20.50 | 1.000 | .976 | 0.024 |
| 21.50 | .992 | .956 | 0.036 |
| 22.50 | .980 | .940 | 0.04 |
| 23.50 | .976 | .907 | 0.069 |
| 24.50 | .968 | .887 | 0.081 |
| 25.50 | .964 | .857 | 0.107 |
| 26.50 | .956 | .826 | 0.13 |
| 27.50 | .944 | .810 | 0.134 |
| 28.50 | .904 | .795 | 0.109 |
| 29.50 | .896 | .775 | 0.121 |
| 30.50 | .888 | .744 | 0.144 |
| 31.50 | .876 | .706 | 0.17 |
| 32.50 | .859 | .671 | 0.188 |
| 33.50 | .843 | .658 | 0.185 |
| 34.50 | .835 | .631 | 0.204 |
| 35.50 | .811 | .620 | 0.191 |
| 36.50 | .787 | .603 | 0.184 |
| 37.50 | .779 | .585 | 0.194 |
| 38.50 | .759 | .561 | 0.198 |
| 39.50 | .743 | .539 | 0.204 |
| **40.50** | **.727** | **.519** | ***0.208** |
| 41.50 | .695 | .503 | 0.192 |
| 42.50 | .663 | .497 | 0.166 |
| 43.50 | .635 | .492 | 0.143 |
| 44.50 | .627 | .486 | 0.141 |
| 45.50 | .614 | .475 | 0.139 |
| 46.50 | .594 | .459 | 0.135 |
| 47.50 | .566 | .444 | 0.122 |
| 48.50 | .558 | .437 | 0.121 |
| 49.50 | .542 | .415 | 0.127 |
| 50.50 | .522 | .402 | 0.12 |
| 51.50 | .502 | .380 | 0.122 |
| 52.50 | .490 | .349 | 0.141 |
| 53.50 | .478 | .331 | 0.147 |
| 54.50 | .466 | .313 | 0.153 |
| 55.50 | .438 | .302 | 0.136 |
| 56.50 | .422 | .285 | 0.137 |
| 57.50 | .418 | .274 | 0.144 |
| 58.50 | .382 | .258 | 0.124 |
| 59.50 | .378 | .243 | 0.135 |
| 60.50 | .357 | .241 | 0.116 |
| 61.50 | .345 | .234 | 0.111 |
| 62.50 | .333 | .230 | 0.103 |
| 63.50 | .317 | .221 | 0.096 |
| 64.50 | .297 | .205 | 0.092 |
| 65.50 | .281 | .192 | 0.089 |
| 66.50 | .273 | .183 | 0.09 |
| 67.50 | .257 | .177 | 0.08 |
| 68.50 | .241 | .166 | 0.075 |
| 69.50 | .233 | .157 | 0.076 |
| 70.50 | .217 | .148 | 0.069 |
| 71.50 | .213 | .139 | 0.074 |
| 72.50 | .209 | .132 | 0.077 |
| 73.50 | .201 | .119 | 0.082 |
| 74.50 | .193 | .110 | 0.083 |
| 75.50 | .181 | .091 | 0.09 |
| 76.50 | .161 | .084 | 0.077 |
| 77.50 | .153 | .077 | 0.076 |
| 78.50 | .124 | .064 | 0.06 |
| 79.50 | .120 | .057 | 0.063 |
| 80.50 | .116 | .051 | 0.065 |
| 81.50 | .108 | .046 | 0.062 |
| 82.50 | .084 | .042 | 0.042 |
| 83.50 | .072 | .040 | 0.032 |
| 84.50 | .064 | .038 | 0.026 |
| 85.50 | .052 | .026 | 0.026 |
| 86.50 | .048 | .026 | 0.022 |
| 87.50 | .044 | .026 | 0.018 |
| 88.50 | .036 | .024 | 0.012 |
| 89.50 | .036 | .020 | 0.016 |
| 90.50 | .036 | .013 | 0.023 |
| 91.50 | .024 | .009 | 0.015 |
| 92.50 | .016 | .009 | 0.007 |
| 93.50 | .012 | .007 | 0.005 |
| 94.50 | .012 | .002 | 0.01 |
| 96.00 | .008 | .000 | 0.008 |
| 100.00 | .004 | .000 | 0.004 |
| 104.00 | .000 | .000 | 0 |
| ROC, Receiver operating curve; *40.5 years of age indicates the highest Youden’s index. ^a^ Youden’s index = (Sensitivity + Specificity) – 1 | | | |
