# Supplementary figures and images for: Predictors of 72-h unscheduled return visits with admission in patients presenting to the emergency department with abdominal pain
Source: Eur J Med Res. 2023 Aug 17;28:288. doi: 10.1186/s40001-023-01256-7 (PMC10433659; doi:10.1186/s40001-023-01256-7)

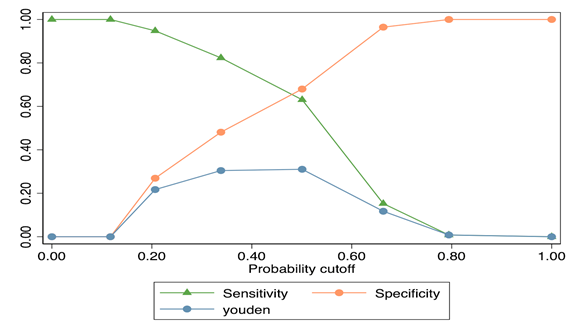

Supplement: Supplementary file 2 — Additional file 2: Optimal cut-off point. [file 40001_2023_1256_MOESM2_ESM.tiff]
